# Restless legs syndrome and tension-type headache: a population-based study

**DOI:** 10.1186/s10194-017-0754-x

**Published:** 2017-04-19

**Authors:** Pil-Wook Chung, Soo-Jin Cho, Won-Joo Kim, Kwang Ik Yang, Chang-Ho Yun, Min Kyung Chu

**Affiliations:** 1 Department of Neurology, Kangbuk Samsung Hospital, Sungkyunkwan University School of Medicine, Seoul, Republic of Korea; 2 Department of Neurology, Dongtan Sacred Heart Hospital, Hallym University College of Medicine, Hwaseong, Republic of Korea; 3 Department of Neurology, Gangnam Severance Hospital, Yonsei University, College of Medicine, Seoul, Republic of Korea; 4 Department of Neurology, Soonchunhyang University College of Medicine, Cheonan Hospital, Cheonan, Republic of Korea; 5 Department of Neurology, Bundang Clinical Neuroscience Institute, Seoul National University Bundang Hospital, Seongnam, Republic of Korea; 6 Department of Neurology, Kangnam Sacred Heart Hospital, Hallym University College of Medicine, 1 Singil-ro, Yeongdeungpo-gu, Seoul, 07441 Republic of Korea

**Keywords:** Restless legs syndrome, Tension-type headache, Anxiety, Depression

## Abstract

**Background:**

Recent studies have shown a significant association between restless legs syndrome (RLS) and primary headache disorders. Nevertheless, information regarding the association between tension-type headache (TTH) and RLS is limited. This study aimed to investigate the association between RLS and TTH in a population-based sample.

**Methods:**

We selected a stratified random population sample of Koreans aged 19–69 years and assessed them using a semi-structured interview designed to identify RLS, headache type, and clinical characteristics of TTH. We determined the prevalence and clinical impact of RLS in participants with TTH.

**Results:**

Of the 2695 participants, 570 (21.2%) and 142 (5.3%) were classified as having TTH and RLS, respectively. Among the 570 individuals with TTH, 113 (19.8%) also met the criteria for probable migraine (PM). The prevalence of RLS was significantly higher among individuals with TTH than among those with non-headache (6.0% vs 3.6%, *p* = 0.018). The prevalence of RLS was significantly higher in subjects with TTH who fulfilled PM criteria than in those with non-headache participants (8.0% vs. 3.6%, *p* = 0.018). However, RLS prevalence in individuals with TTH who did not fulfil PM criteria did not differ from that of participants with non-headache (5.5% vs. 3.6%, *p* = 0.063). TTH participants with RLS had higher visual analogue scale scores for headache intensity (5.1 ± 2.0 vs. 4.3 ± 1.8, *p* = 0.038), and higher prevalence of anxiety (20.6% vs. 8.8%, *p* = 0.022) and depression (14.7% vs. 3.5%, *p* = 0.002) than TTH participants without RLS. Multivariable analyses revealed that headache aggravation by movement (odds ratio [OR] = 2.4, 95% confidence interval [CI] = 1.1–5.2) and depression (OR = 3.5, 95% CI = 1.1–11.4) were significant indicators of RLS among individuals with TTH.

**Conclusions:**

The prevalence of RLS was higher among individuals with TTH than among those with non-headache. Some clinical presentations varied in accordance with the presence of RLS among participants with TTH.

**Electronic supplementary material:**

The online version of this article (doi:10.1186/s10194-017-0754-x) contains supplementary material, which is available to authorized users.

## Background

Tension-type headache (TTH) is the most common type of primary headache disorder and is the third most common disease in the world [1]. TTH has been considered a non-serious disorder compared to migraine. However, it is a major health problem and leads to a severe socioeconomic burden owing to its high prevalence and comorbidities such as anxiety, depression, temporo-mandibular disorders, fibromyalgia, and obstructive sleep apnoea [2–6]. Patients with TTH with comorbidities have more severe symptoms than those without comorbidities [2]. Therefore, identification of the associated comorbidities of TTH is important for better management of TTH and may reduce the burden of the disease.

Restless legs syndrome (RLS) is a sleep-related sensory-motor disorder characterized by unpleasant feelings in the legs, especially during rest or at bedtime. The unpleasant feelings are relieved by voluntary leg movement [7]. Previous population-based and clinic-based studies have consistently reported a significant association between RLS and migraine [8–14].

The mechanism for the association between migraine and RLS is still uncertain. Dopamine and iron dysregulation have been proposed to be mechanism both for migraine and RLS [7, 15–17]. Therefore, they were considered as mechanisms for the comorbidity of two disorders. Furthermore, genetic associations between RLS and migraine have been suggested [18]. Although numerous reports have suggested a close association between migraine and RLS, population-based information regarding the association between RLS and TTH, which is the most common type of headache, is limited. Nevertheless, a few studies have suggested the presence of significant associations between RLS and TTH. A population-based report indicates that RLS has a significant association with non-migraine headache [14]. A health-insurance database study in Taiwan revealed that individuals with TTH have an increased risk for developing RLS [19].

We hypothesized that RLS and TTH would have a significant association in a general population-based sample. The Korean Headache-Sleep Study (KHSS) is a cross-sectional nationwide population-based survey on sleep and headache in Korean adults aged 19–69 years and may provide an opportunity for us to assess the association between RLS and TTH. Therefore, we estimated the prevalence of TTH and RLS in a Korean general population-based sample, compared the association between TTH and RLS in comparison with individuals with non-headache, and assessed the clinical impact of RLS in individuals with TTH using data from the KHSS.

## Methods

### Survey

The KHSS, used in this study, was approved by the institutional review board and ethics committee of Hallym University Sacred Heart Hospital and written informed consent was obtained from all participants. Details of the KHSS have previously been described [14, 20]. Briefly, we used a 2-stage clustered random sampling method to obtain a sample proportional to the population distribution of Korea, with the exception of Jeju Island. Participants were stratified by age, sex, and occupation. We informed candidates that the survey topic was general health rather than headache and sleep to avoid interest bias. All interviewers were employees of Gallup Korea. The survey was conducted between November 2011 and January 2012 via personal visits and interviews, using a questionnaire that was designed to identify headache type, anxiety, depression, and RLS.

### Diagnosis of TTH

Diagnosis of TTH was based on the criteria for infrequent TTH (code 2.1) of the third edition beta version of the International Classification of Headache Disorders (ICDH-3 beta) [21]. Participants were considered to have TTH if they met criteria B through E (B: lasting from 30 min to 7 days; C: at least two of the four typical headache characteristics [i.e. bilateral pain, non-pulsating quality, mild-to-moderate pain intensity, and no aggravation by routine physical activity]; D: attacks associated with both of the following: no nausea or vomiting, not having both photophobia and phonophobia, and E: not better accounted for by another ICHD-3 diagnosis). The questionnaire used in the present study had 75.0% sensitivity and 88.2% specificity when compared to physicians’ diagnoses [22]. We did not apply the frequency criterion (criterion A) in the diagnosis of TTH. Thus, patients with TTH evaluated in this study included those with infrequent TTH (code 2.1), frequent TTH (code 2.2), and chronic TTH (code 2.3). According to ICHD-3 beta, if a participant’s headache met the criteria for both TTH and probable migraine (PM), he or she was considered to have TTH [21].

### Diagnosis of RLS

Diagnosis of RLS was based on the Korean version paradigm of questions for the epidemiology studies of RLS [7, 23]. The paradigm for question for the epidemiology studies for RLS was based on the International Restless Legs Syndrome Study Group criteria published in 2003 [7]. The Korean version paradigm of question for the epidemiology studies of RLS was composed of 3 questions: (1) ‘Do you have unpleasant sensations in your legs combined with an urge or need to move your legs?’ (2) ‘Do these feelings occur mainly or only at rest and do they improve with movement?’ (3) ‘Are these feelings worse in the evening or night than in the morning?’. The Korean version paradigm of question for the epidemiology studies for RLS was validated with 85.3% sensitivity and 89.5% specificity by comparing doctors’ diagnosis of RLS [23].

### Assessment of anxiety and depression

Anxiety was measured using the Goldberg Anxiety Scale (GAS) [24]. The Korean version of GAS has 82.0% sensitivity and 94.4% specificity for the diagnosis of anxiety and has previously been validated [25]. To diagnose depression, the Patient Health Questionnaire-9 was used [26]. Participants with scores of 10 or higher on this measure were considered to have depression. The Korean version of the Patient Health Questionnaire-9 has 81.1% sensitivity and 89.9% specificity [27]. To assess headache intensity and the impact of the headache, we used the Visual Analogue Scale (VAS) and the Headache Impact Test-6, respectively.

### Statistical Analyses

The Kolmogorov-Smirnov test was used to confirm the normality of the distributions. Following confirmation of a normal distribution, we used Student’s t-tests or Analysis of Variance to compare continuous variables. In cases of non-normal distribution, we used Mann–Whitney *U* tests or Kruskal-Wallis tests. Categorical variables were compared using Chi-square tests.

We investigated factors contributing to RLS among individuals with TTH using univariable and multivariable analyses. To perform univariable analyses, we considered factors with significant differences between TTH participants with RLS and those without RLS. To perform multivariable analyses, we developed 4 models for the association between RLS and TTH. Model 1 included sociodemographic variables (age, sex, size of residential area, and education level) and headache aggravation by movement. Model 2 added anxiety as a variable to Model 1. In Model 3, we added depression to Model 1. The final model, Model 4, incorporated sociodemographic variables, aggravation by movement, and anxiety and depression. A significance level of *p* < 0.05 was used for all analyses. Analyses were conducted using the Statistical Package for the Social Sciences 22.0 (SPSS 22.0; IBM, Armonk, NY).

Similar to most survey sampling designs, there were missing data (resulting from non-response) for several variables. All of the reported results are based on the available data. As such, the total numbers for some variables diverge from 2695 because of missing data for that particular variable. Imputation techniques were not used because we wanted to minimize non-response effects [28].

## Results

### Survey

**Fig. 1:**
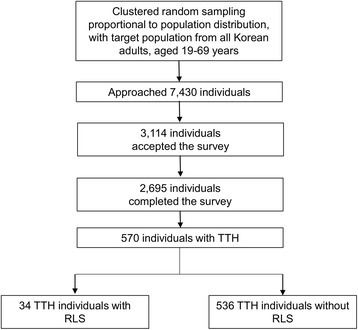
Flowchart depicting the participation of subjects in the Korean Headache-Sleep Study. *RLS,* restless legs syndrome; *TTH,* tension-type headache

Interviewers approached 7430 individuals. Of the 7430 individuals, 3114 agreed to participate in this survey (rejection rate, 58.1%). Of these participants, 2695 completed the survey (cooperation rate, 36.3%; Fig. 1). Distributions of age, sex, size of residential area, and education level were not significantly different between our survey participants and the general population of Korea (Additional file 1: Table S1).

### Prevalence of TTH and RLS

Of the 2695 participants, 570 (21.2%) were considered to have TTH and 1422 (52.8%) were classified as non-headache participants. Of the 570 participants who were classified as TTH, 14 participants (2.5%) were classified as chronic TTH (code 2.3), 229 (40.2%) as infrequent episodic TTH (code 2.1), and 327(57.3%) as frequent episodic TTH (code 2.2). Among the individuals with TTH, 113 (19.8%) also met the criteria for PM. The prevalence of TTH was not significantly affected by age, sex, or educational level. The prevalence of TTH was higher in rural areas compared to large cities and small-to-medium cities (Table 1). One hundred and forty-two (5.3%) participants were classified as having RLS. Restless legs syndrome was more prevalent in women and participants with lower educational level (middle school or less) than among men and those with higher educational level. The prevalence of RLS had a trend toward an increase with increasing age (Additional file 1: Table S1). Sociodemographic characteristics of individuals with TTH and non-headache are summarized in Table 1. Women and participants living in rural areas were more common among individuals with TTH than among those with non-headache. However, distributions of age and educational level were not significantly different between the two groups.

**Table 1:** Sociodemographic characteristics and RLS status in participants with TTH and participants with non-headache

	All participants,	Participants with TTH, *N* (%)	Participants with non-headache, *N* (%)	*P* ^a^
	N			
Sex				<0.001
Male	1345	268 (19.9)	838 (62.3)	
Female	1350	302 (22.3)	584 (43.3)	
Age				0.405
19–29	542	119 (22.0)	286 (52.8)	
30–39	604	127 (21.0)	293 (48.5)	
40–49	611	131 (21.4)	295 (48.3)	
50–59	529	107 (20.2)	303 (57.3)	
60–69	409	86 (21.0)	245 (59.9)	
Size of the residential area				0.009
Large city	1248	251 (20.1)	647 (51.8)	
Small-to-medium city	1186	243 (20.5)	650 (54.8)	
Rural area	261	76 (29.1)	125 (47.9)	
Education level				0.345
Middle school or lower	393	96 (24.5)	208 (52.4)	
High school	1208	247 (20.5)	646 (53.5)	
College or higher	1068	223 (20.9)	551 (51.6)	
No response	26	4 (15.4)	19 (73.1)	
RLS	142	34 (6.0)	51 (3.6)	0.018

^a^Comparison between participants with tension-type headache and those without headache. *TTH* Tension-type headache, *RLS* Restless legs syndrome

### Prevalence of anxiety and depression

Two-hundred and sixty-eight (9.9%) and 116 (4.3%) participants were classified as having anxiety and depression, respectively. The prevalence of anxiety (9.5% vs. 5.3%, *p* = 0.001) and depression (4.2% vs. 1.8%, *p* = 0.001) were significantly higher among individuals with TTH than among those with non-headache.

### Prevalence of RLS in participants with TTH

**Fig. 2:**
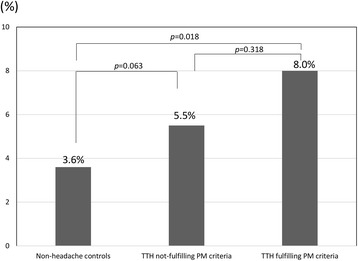
Prevalence of RLS among individuals with non-headache, those with TTH fulfilling PM criteria, and those with TTH not fulfilling PM criteria. *PM,* probable migraine; *RLS,* restless legs syndrome; *TTH,* tension-type headache

Among the 570 individuals with TTH, 34 (6.0%) were classified as having RLS. The prevalence of RLS was significantly higher among individuals with TTH than among individuals with non-headache (6.0% vs 3.6%, *p* = 0.018). Among the 113 participants with TTH who fulfilled PM criteria, 9 participants (8.0%) were classified as having RLS. The prevalence of RLS among participants with TTH who also fulfilled PM criteria was not significantly different from that among those with TTH not fulfilling PM criteria (8.0% vs. 5.5%, *p* = 0.318). The prevalence of RLS in participants with TTH not fulfilling PM criteria did not differ from that of individuals with non-headache (5.5% vs. 3.6%, *p* = 0.063). Nevertheless, the prevalence of RLS among individuals with TTH fulfilling PM criteria was significantly higher than that among those with non-headache (8.0% vs. 3.6%, *p* = 0.018; Fig. 2). Prevalence of RLS was analysed according to headache frequency. Prevalence of RLS in participants with TTH with 1-10 attacks per month (5.9%, *p* = 0.861) and participants with TTH with >10 attacks per month (13.6%, *p* = 0.116) was numerically higher compared to that of those with <1 attack per month (5.5%). However, it did not reach statistical significance.

We also investigated the prevalence of RLS in individuals with TTH grouped on the basis of anxiety and depression status (Table 2). The prevalence of RLS was significantly higher among TTH participants with anxiety than among those without anxiety (13.0% vs. 5.2%, *p* = 0.022). The prevalence of RLS was significantly higher among TTH subjects with depression than among those without depression (20.8% vs. 5.3%, *p* = 0.002).

**Table 2:** Demographics, headache characteristics, and associated symptoms in subjects with TTH according to the presence of RLS

	TTH subjects with RLS	TTH subjects without RLS	
			*P*
	*N* = 34	*N* = 536	
Demographics			
Mean age ± SD (years)	46.4 ± 13.3	42.5 ± 13.7	0.108
Women, *N* (%)	20 (58.8)	282 (52.6)	0.482
Headache characteristics			
Bilateral pain, *N* (%)	23 (67.6)	351 (65.5)	0.797
Non-pulsating quality, *N* (%)	16 (47.1)	211 (39.4)	0.374
Mild-to-moderate severity, *N* (%)	34 (100.0)	526 (98.1)	0.422
Non-aggravation by movement, *N* (%)	22 (64.7)	429 (80.0)	0.033
Associated symptoms			
Photophobia, *N* (%)	5 (14.7)	41 (7.6)	0.143
Phonophobia, *N* (%)	9 (26.5)	172 (32.1)	0.495
Osmophobia, *N* (%)	7 (20.6)	88 (16.4)	0.527
Mood			
Anxiety	7 (20.6)	47 (8.8)	0.022
Depression	5 (14.7)	19 (3.5)	0.002
Headache frequency per month	2.3 ± 3.5	1.9 ± 4.9	0.054
VAS score for headache intensity	5.1 ± 2.0	4.3 ± 1.8	0.038

*RLS* Restless legs syndrome, *TTH* Tension-type headache, *VAS* Visual Analogue Scale

### Demographics and clinical presentations of participants with TTH according to RLS status

The demographics and clinical presentations of participants with TTH according to the presence of RLS are summarized in Table 2. Demographic variables, headache characteristics (except for non-aggravation by movement), and accompanying symptoms were not significantly different between TTH subjects with RLS and those without RLS. Visual Analogue Scale scores for headache intensity were significantly higher among TTH subjects with RLS than among those without RLS. Headache frequency was marginally different between the two groups, although this difference was not significant. The prevalence of anxiety and depression were significantly higher among participants with TTH with RLS than in those without RLS.

### Factors contributing to RLS among participant with TTH

Univariate analyses revealed that headache aggravation by movement (odds ratio [OR] = 2.2, 95% confidence interval [CI] = 1.1–4.6), anxiety (OR = 2.7, 95% CI = 1.2–6.5), and depression (OR = 4.7, 95% CI = 1.6–13.5) are associated with an increased risk for RLS. Multivariable analyses including sociodemographic variables and aggravation by movement (Model 1) indicated that aggravation by movement leads to an increased OR for RLS (OR = 2.3, 95% CI = 1.1–4.8). In Model 2 (including sociodemographic variables, aggravation by movement, and anxiety), aggravation by movement (OR = 2.3, 95% CI = 1.1–4.9) and anxiety (OR = 3.4, 95% CI = 1.3–8.8) had significant effects on RLS status. In Model 3 (including sociodemographic variables, aggravation by movement, and depression), aggravation by movement (OR = 2.4, 95% CI = 1.1–5.2) and depression (OR = 4.9, 95% CI = 1.6–15.0) led to increased ORs for RLS. Model 4 (including sociodemographic variables, aggravation by movement, anxiety, and depression) revealed that aggravation by movement (OR = 2.4, 95% CI = 1.1–5.2) and depression (OR = 3.5, 95% CI = 1.1–11.4) have significant effects on RLS status (Table 3).

**Table 3:** Univariate and multivariable logistic regression analyses of RLS among individuals with tension-type headache (*N* = 570)

	Univariable analyses	Multivariable analyses			
		Model 1^a^	Model 2^b^	Model 3^c^	Model 4^d^
	OR (95% CI)	OR (95% CI)	OR (95% CI)	OR (95% CI)	OR (95% CI)
Aggravation by movement	2.2 (1.1–4.6)	2.3 (1.1–4.8)	2.3 (1.1–4.9)	2.4 (1.1–5.2)	2.4 (1.1–5.2)
Anxiety	2.7 (1.2–6.5)		3.4 (1.3–8.8)		2.6 (0.9–7.0)
Depression	4.7 (1.6–13.5)			4.9 (1.6–15.0)	3.5 (1.1–11.4)

*OR* odds ratio, *CI* confidence interval

^a^Including sociodemographic variables (age, sex, size of residential area, and educational level) and aggravation by movement

^b^Including sociodemographic variables, aggravation by movement, and anxiety

^c^Including sociodemographic variables, aggravation by movement, and depression

^d^Including sociodemographic variables, aggravation by movement, anxiety, and depression

## Discussion

The main findings of the present study are as follows: 1) The prevalence of TTH and RLS are 21.1% and 5.3%, respectively, in the Korean general population sample used; 2) The prevalence of RLS among subjects with TTH was significantly higher than among participants with non-headache; and 3) TTH participants with RLS had more severe headache intensity and higher prevalence of anxiety and depression than those without RLS.

While a significant association between RLS and migraine has been consistently reported, information regarding the association between RLS and TTH, which is another common primary headache, is limited. Recently, a retrospective cohort study using a health insurance database suggested that patients with TTH had an increased risk of developing RLS [19]. Nevertheless, the study did not provide cross-sectional evidence for an association between RLS and TTH, identified TTH and RLS cases on the basis of diagnostic codes rather than diagnostic criteria and did not assess the impact of RLS on the clinical presentation of TTH. Here, we identified RLS and TTH cases on the basis of validated criteria and found that RLS was more prevalent among individuals with TTH than among those with non-headache in a general population-based sample. In addition, we found that participants with TTH with RLS had more severe headache intensity than those with TTH without RLS.

In the present study, TTH individuals with anxiety and depression had higher RLS prevalence than those without anxiety and depression. Multivariable regression analyses revealed that depression is an independent predictor of RLS. These findings are concurrent with those of previous studies of the association between RLS and mood symptoms. Individuals with anxiety or depression have been shown to have a higher risk of RLS and vice versa [29–31]. Anxiety and depression are also common comorbid conditions of TTH [3]. Therefore, it is plausible that mood symptoms, such as anxiety and depression, are important factors linking RLS to TTH. Further studies are required among TTH subjects regarding mood symptoms and RLS to elucidate the associations among RLS, mood symptoms, and TTH.

We classified 113 participants fulfilling both TTH and PM criteria as subjects with TTH in accordance with the general rule of ICHD-3 beta. Although RLS prevalence in participants with TTH fulfilling PM criteria did not significantly differ from that in those with TTH not fulfilling PM criteria, RLS prevalence in participants with TTH fulfilling PM criteria was significantly higher than in those with non-headache. However, RLS prevalence among participants with TTH not fulfilling PM criteria was not different from that in participants with non-headache (Fig. 2). Among migrainous features, aggravation by movement was more frequently found in TTH participants with RLS than in those without RLS (Table 2). Univariable and multivariable analyses indicated that headache aggravation by movement is an independent predictor of RLS among participants with TTH (Table 3). These findings were concurrent with previous findings that the frequency of RLS may increase with an increase in the number of migrainous symptoms among migraineurs [8]. However, other migrainous features, such as unilateral pain, pulsating quality, moderate-to-severe headache intensity, photophobia, and phonophobia were not significantly different between individuals with and those without RLS in the present study. Further studies including various headache populations will validate the association between migrainous features and RLS among headache sufferers.

The 1-year TTH prevalence rate (21.2%) in the present study was somewhat lower than that previously observed in Western countries (20–78%) [32]. The 1-year prevalence of TTH in Asian countries ranges from 10.3 to 33.3% in most studies of Asian populations, which is somewhat lower than those reported in studies of European and North American countries [32, 33]. RLS prevalence (5.3%) in the present study was similar to those reported in previous studies from Korea and other Asian countries, which range from 1.8 to 8.3%. Similar to TTH, the reported RLS prevalence in Asian populations is lower than that observed in Western countries [5, 23, 30, 34]. Therefore, the prevalence of RLS and TTH in the present study is similar to the reported prevalence in Asian countries. The similarities between the prevalence of RLS and TTH in the present study and in previous reports from Asian countries suggest that our study properly evaluated RLS and TTH.

Restless leg syndrome has long been considered to be caused by dopaminergic system dysfunction and disturbed iron metabolism [16, 35]. Unbalanced level of dopaminergic neurotransmitter might also be involved in migraine pathogenesis and dysfunctional iron metabolism in the brain of migraine has been suggested [15, 17, 18]. Therefore, it is plausible that some underlying migraine characteristics may be a substrate for linking TTH and RLS in consideration of more strong association between TTH fulfilling PM criteria and RLS than between TTH not fulfilling PM criteria and RLS in the present study.

In the present study, depression was significant contributing factor for RLS among individuals with TTH in univariable and multivariable analyses. Association of depression and RLS has been documented in population-based studies [34, 36]. Dopaminergic dysfunction was noted in depression [37]. It also has been considered as a key mechanism of RLS as mentioned above [35]. Dopaminergic dysregulation in TTH has been proposed in previous studies [38, 39]. Accordingly, dopaminergic dysfunction could be a mechanism for connecting depression and RLS among individuals with TTH. Further experimental and clinical studies were needed for the association of depression, RLS and TTH.

There are several limitations to the present study. Firstly, we diagnosed RLS by using the Korean version paradigm of questions for the epidemiology studies of RLS based on participant’s report [23]. Therefore, some conditions similar to RLS may have been included. Akathisia, meralgia paresthetica, peripheral neuropathy, and nocturnal cramping can mimic RLS. Nevertheless, the Korean version paradigm of questions for the epidemiology studies of RLS showed high sensitivity and specificity for the diagnosis of RLS when comparing doctors’ diagnosis of RLS [7, 23]. Secondly, we did not assess the severity of RLS owing to limitations on questionnaire length. An attempt to quantitatively correlate RLS and headache may add more insight to the association between TTH and RLS. Thirdly, although the current study used a population-based sample with low sampling error, its statistical power was limited in terms of examining the subgroups of interest. In other words, the lack of significant findings in the subgroup analyses might be the result of the limited sample size.

Our study has several strengths. Firstly, we used the data of KHSS which was based on clustered random sampling proportional to the Korean population distribution with low sampling error. This condition allowed us to precisely investigate the association between RLS and TTH in a population-base setting. Secondly, we investigated anxiety and depression, which are common comorbidities among individuals of RLS and assessed the effect of anxiety and depression in the association between RLS and TTH. Thirdly, we assessed the prevalence of RLS among individuals with TTH according to migrainous features.

## Conclusions

In conclusion, the prevalence of RLS among individuals with TTH was significantly higher than that among those with non-headache in a population-based sample in Korea. TTH participants with RLS had higher VAS scores for headache intensity than those without RLS. Headache aggravation by movement and depression were significant indicators of the presence of RLS among individuals with TTH. Our findings may provide a better understanding of the comorbidity between RLS and TTH.

## Additional file

Additional file 1: Table S1. Sociodemographic characteristics of survey participants, the total Korean population, and cases identified as having TTH and RLS. (DOCX 20 kb)

## Abbreviations

CI: Confidence interval
GAS: Goldberg anxiety scale
ICHD: International classification of headache disorders
IRLSSG: International restless legs syndrome study group
KHSS: Korean headache-sleep study
OR: Odds ratio
PM: Probable migraine
RLS: Restless legs syndrome
TTH: Tension-type headache
VAS: Visual analogue scale
