# Supplementary material for: Restless legs syndrome and tension-type headache: a population-based study
Source: J Headache Pain. 2017 Apr 19;18(1):47. doi: 10.1186/s10194-017-0754-x (PMC5397394; doi:10.1186/s10194-017-0754-x)
Supplement: Additional file 1: Table S1. — Sociodemographic characteristics of survey participants, the total Korean population, and cases identified as having TTH and RLS. (DOCX 20 kb) [file 10194_2017_754_MOESM1_ESM.docx]

Additional file 1: Table S1. Sociodemographic characteristics of survey participants, the total Korean population, and cases identified as having TTH and RLS.

|  |  | Survey participants |  | Total population | P | Tension-type headache | P | Restless legs syndrome | P |
| --- | --- | --- | --- | --- | --- | --- | --- | --- | --- |
|  |  | N (%) |  | N (%) |  | N, % (95% CI) |  | N, % (95% CI) |  |
| Sex |  |  |  |  |  |  |  |  |  |
|  | Men | 1345 (49.3) |  | 16,357,919 (50.6) | 0.855 | 268, 19.9 (17.8-22.0) | 0.120 | 56, 4.1 (3.0-5.1) | 0.010 |
|  | Women | 1350 (50.7) |  | 15,998,828 (49.4) |  | 302, 22.3 (20.1-24.6) |  | 86, 6.4 (5.1-7.7) |  |
| Age |  |  |  |  |  |  |  |  |  |
|  | 19–29 | 542 (20.5) |  | 7,717,947 (22.2) | 0.917 | 119, 22.0 (18.5-25.5) | 0.971 | 12, 2.2 (1.0-3.4) | <0.001 |
|  | 30–39 | 604 (21.9) |  | 8,349,487 (24.0) |  | 127, 21.0(17.8-24.3) |  | 34, 5.6 (3.7-7.4) |  |
|  | 40–49 | 611 (23.1) |  | 8,613,110 (24.8) |  | 131, 21.4 (18.2-24.7) |  | 20, 3.3 (1.9-4.7) |  |
|  | 50–59 | 529 (18.9) |  | 6,167,505 (17.7) |  | 107, 20.2 (16.8-23.7) |  | 35, 6.5 (4.4-8.7) |  |
|  | 60–69 | 409 (15.6) |  | 3,934,666 (11.3) |  | 86, 21.0 (17.1-25.0) |  | 41, 10.2 (7.3-13.1) |  |
| Size of residential area | |  |  |  |  |  |  |  |  |
|  | Large city | 1248 (46.3) |  | 1,5606,652 (48.2) | 0.921 | 251, 20.1 (17.9-22.4) | 0.004 | 66, 5.3 (4.1-6.5) | 0.042 |
|  | Small-to-medium city | 1186 (44.0) |  | 1,4106,687 (43.6) |  | 243, 20.5 (18.2-22.8) |  | 58, 4.9 (3.7-6.1) |  |
|  | Rural area | 261 (9.7) |  | 264,307 (8.2) |  | 76, 29.1 (23.6-34.7) |  | 18, 6.8 (3.7-9.8) |  |
| Education level | |  |  |  |  |  |  |  |  |
|  | Middle school or lower | 393 (14.9) |  | 6,147,782 (19.0) | 0.752 | 96, 4.5 (20.1-28.7) | 0.327 | 41, 10.5 (7.5-13.5) | <0.001 |
|  | High school | 1208 (44.5) |  | 14,172,255 (43.8) |  | 247, 20.5 (18.2-22.7) |  | 62, 5.1 (3.8-6.3) |  |
|  | College or higher | 1068 (39.6) |  | 1,2036,710 (37.2) |  | 223, 20.9 (18.4-23.3) |  | 38, 3.6 (2.5-4.7) |  |
|  | No response | 26 (9.6) |  |  |  | 4, 15.4 (0.5-30.2) |  | 1, 3.8 (0.0-11.8) |  |
| Total |  | 2695 (100.0) |  | 32,356,747 (100.0) |  | 570, 21.2 (19.6-22.7) |  | 142, 5.3 (4.4-6.1) |  |

Variables are presented as number (%) or number, % (95% confidence interval). *N,* number; *CI,* confidence interval
